# Palliative care consultation teams in long-term care: a descriptive retrospective cohort study

**DOI:** 10.1186/s12904-025-01716-3

**Published:** 2025-03-22

**Authors:** Helen Tam-Tham, Nadine Persaud, Amit Arya

**Affiliations:** 1 Department of Family Medicine, University of Calgary, Calgary, Alberta Canada; 2 Department of Oncology, University of Calgary, Calgary, Alberta Canada; 3 Kensington Research Institute, Toronto, Ontario Canada; 4 Division of Palliative Care, Department of Family and Community Medicine, University of Toronto, Toronto, Ontario Canada

**Keywords:** Palliative care, Long-term care, Referral and consultation, Cohort studies

## Abstract

**Purpose:**

Given the wide prevalence of advanced illness and frailty among residents in long-term care (LTC), a palliative approach to care can support comfort and quality of life. Yet, significant gaps exist with the provision of palliative care in LTC settings. We aim to describe a palliative care consultation team designed to address this need.

**Methods:**

A single-centre retrospective cohort study was conducted at a LTC home in Toronto, Ontario, Canada. We included residents referred to the palliative care consultation team between February 1, 2021, to February 1, 2023, with at least six-months of follow-up time. We used a descriptive quantitative approach to examine access to the palliative care consultation team, changes to advance care plans, and hospital transfers.

**Results:**

Eighty-seven residents were referred and seen by the palliative care consultation team. The mean age was 85 years, 71.3% were female, and 48.3% had three to four comorbidities. Most residents were seen once (55.2%). Among residents that died (*n* = 53), 41.5% were referred with greater than three months of survival time. Among residents that had advance care plans documenting “Transfer to Hospital” (*n* = 41) and “Full Code” status (*n* = 17), 53.7% adjusted to “Do Not Transfer” and 76.5% to “Do Not Resuscitate” orders, respectively. The hospitalisation rate was one per 1000 resident-year.

**Conclusions:**

At this LTC home, palliative care consultation teams represented an important service to improve the provision of palliative care particularly around facilitating advance care planning discussions. The findings of this study may inform further research on palliative care interventions for LTC residents.

## Background

The number of Canadians aged 65 and older is projected to increase significantly in the coming decades. By 2036, this age group will constitute 25% of the population. Further, the number of Canadians aged 80 and older is expected to nearly triple to over 3 million by 2036 and almost quadruple to more than 5 million by 2061 [1]. This poses a number of challenges on the healthcare system both from a resource and capacity standpoint, and is contributing to an increased demand for long-term care (LTC) [2]. The LTC residents of today live with multiple chronic illnesses and severe disabilities, leading to higher complexity and acuity, as well as reduced life expectancy [3, 4]. Therefore, there is a recognized need for palliative care in LTC settings to support the needs of this population. Palliative care in LTC should not be restricted to end-of-life care. Rather, a palliative care approach should be integrated early in the course of an illness to provide support throughout its disease trajectory [4]. 

Between 2012 and 2022, 19% of LTC residents in Canada received some form of palliative care in the last year of life. Additionally, 28% of LTC residents who died in hospital were admitted specifically for end-of-life care [3]. Given that LTC homes have access to around-the-clock nursing care, medical care, and social care, residents should be able to die in place. However this is not often possible, as LTC homes have inadequate staff, a lack of resources and equipment, as well as a need for palliative care education [3, 5–7]. Overall, there is a need to improve palliative and end-of-life care within the LTC sector. Enhancing these services will not only support the quality of life for residents but also reduce unnecessary hospital transfers for end-of-life care.

Palliative care providers have a unique role in supporting people living with advanced frailty and multiple life-limiting illnesses. As part of a framework for implementing and improving palliative care in LTC [8], palliative care teams can take a critical role in enhancing early identification and management of symptoms; provide psychosocial and spiritual support; as well as enhance shared decision making to ensure that care is aligned with one’s wishes. In LTC, previous research has demonstrated that palliative care consultation teams can reduce depression and emergency department visits [9]. Palliative care consultations in LTC are associated with lower rates of potentially burdensome transitions to hospital, especially at the end-of-life [10]. The aim of this paper is to describe the integration of a palliative care consultation team in a single LTC home within a major metropolitan centre in Ontario, Canada. Study objectives include describing the study cohort, examining access to the palliative care consultation team, reviewing changes to advance care plans, and measuring hospital transfer rates.

## Methods

### Study population and setting

We conducted a retrospective cohort study at Kensington Gardens, a not-for-profit LTC home in Toronto, Ontario. The LTC home provides 350 beds with around-the-clock nursing care and interdisciplinary support. The LTC home census (i.e., occupancy rate) was above 97% during the study period. All residents who were referred to the palliative care consultation program were included in the study. The accrual period was for a two-year duration from February 1, 2021, to February 1, 2023, and residents were followed for at least a six-month period until the study end date of August 1, 2023.

### Palliative care consultation team

The palliative care consultation team involved two palliative care physicians and a social worker with a doctoral degree in palliative care. They provided on-site consultations once a week. The consultation team worked collaboratively with the LTC interdisciplinary team members including the most responsible physician, nurse practitioners, nurses, personal support workers, social workers, and other LTC staff to support implementation and continuity of care. The palliative care consultation provided direct clinical care with identification and optimization of pain and symptom management, psychosocial and spiritual support, advance care planning, goals of care discussions, and end-of-life care. Palliative care referrals could be initiated by the LTC interdisciplinary staff members and could be requested by residents and their care partners for any of these domains of palliative care. The palliative care consultation team aimed to see all referrals in-person for their initial consultation. The consultation team continued to provide follow-up support in-person or virtually by telephone or video as needed.

Prior to and after LTC residents were assessed by the palliative care consultation team, the team debriefed with front-line LTC staff to learn about the residents’ care needs and share the results of the assessment. The palliative care consultation team also provided capacity-building at the LTC home. This included interdisciplinary case discussions and formal educational sessions to enhance LTC staff, resident, and care partner knowledge in palliative care principles. These capacity building initiatives, along with the direct clinical care provided, aimed to ensure acceptance of the palliative care consultation team for LTC residents when required.

### Cohort characteristics at the time of palliative care consultation

Data on age, gender, primary language spoken, marital status, primary life-limiting diagnosis, and comorbidities at the date of the initial palliative care consultation were collected retrospectively. We collected data on comorbidities at the time of consultation, including cardiovascular-related diseases such as atrial fibrillation, diabetes, hypertension, chronic kidney disease, heart failure, peripheral vascular disease, and stroke or transient ischemic attack. We collected information on chronic pain and mental health conditions including alcohol misuse, depression, dementia, and schizophrenia. We also collected other clinically important comorbidities such as asthma, chronic pulmonary disease, severe constipation, inflammatory bowel disease, rheumatoid arthritis, and cancers of lymphoma, metastatic, and non-metastatic nature [11]. 

As a measure of function, the Palliative Performance Scale (PPS) was determined at the time of the first palliative care consultation. Using the PPS, 50% is mainly sit/lie, 40% is mainly in bed, 30% is bed bound, 20% with minimal intake, and 10% with mouth care only [12]. The most recent CHESS (Changes in Health, End-stage Disease and Signs and Symptoms) score prior to the initial palliative care consultation was also collected for the study. CHESS scores are based on the interRAI assessment data, which is collected on a quarterly basis and determined by the clinical team at the home. CHESS measures severity of frailty and overall health instability to identify residents at risk of serious decline. CHESS scores range from 0 with no health instability to 5 with very high health instability [13]. Sociodemographic, comorbidity, function, and CHESS data were extracted from the electronic health record via chart review.

### Advance care planning

During the initial palliative care consultation, advance care planning discussions may result in a change in code status (i.e. “Full Code” versus “Do Not Resuscitate”) and future wishes for hospital transfer (i.e. “Transfer to Hospital” versus “Do Not Transfer”). The discussion would occur with a resident if capable, or their substitute decision maker if incapable. In Ontario, all residents legally have a designated substitute decision maker by hierarchy determined by the Health Care Consent Act, which is usually their closest living relative. Substitute decision makers can also be legally appointed. If there are no relatives or legally appointed substitute decision makers, then the Public Guardian and Trustee can act as the substitute decision maker [14]. 

### Hospital transfers rates and end-of-life care

Hospital transfers were defined as transfer out of LTC to hospital for a stay of at least 24 h after the first palliative care consultation. This data was collected retrospectively from the electronic medical record, which includes the dates of admission to LTC, transfers in and out of LTC to hospital for at least 24 h, and the date of death. End-of-life care is generally defined by the last days to short months of life, i.e. less than 90 days of life [15]. 

### Data collection and analysis

All data were collected retrospectively from the electronic medical health record at the LTC home. Descriptive statistics were used to describe the residents who received palliative care consultations. We reported data in frequency (percentage), mean (standard deviation or SD), or median (interquartile range or IQR between 25th and 75th percentiles). We calculated the hospitalisation rate per 1000 resident-years. For confidentiality purposes, we did not report small cell sizes (*n* ≤ 6). Data analyses were conducted with Microsoft Excel software. The University of Toronto Research Ethics Board granted approval and waiver of patient consent on Oct 17, 2022 (#00043339).

## Results

Eighty-seven residents were referred to the palliative care consultation team in the LTC home. The median follow-up was 231 (IQR 47–424) days. The mean age was 85 years (SD 9.1). Many residents were between 80 and 90 years old, female (71.3%), had English as their primary language (72.4%), and were widowed (54.0%). The most common primary life-limiting diagnosis was dementia (50.6%) and frailty (17.2%). Many residents had 3 to 4 comorbidities (48.3%). The most common comorbidities were hypertension (59.8%), dementia (58.6%), and depression (40.2%). Most residents had a CHESS score of 0 (no health instability) to 2 (low health instability), and only 10.3% had a CHESS score of 4 or 5 (high or very high instability). At the time of their first palliative care consultation, many residents had a PPS of 30% (48.3%). The most common substitute decision makers were children (62.1%). See Table 1 for details.

**Table 1:** Characteristics of long-term care residents at the time of palliative care consultation

Baseline characteristic	LTC residents (*n* = 87)
**Age (years)**,** mean (SD)**	85 (9.1)
**Age categories**	
60 to < 80	24 (27.6)
80 to < 90	35 (40.2)
90 to < 100	28 (32.2)
**Gender**	
Female	62 (71.3)
Male	25 (28.7)
**Primary language**	
English	63 (72.4)
Portuguese	7 (8.0)
Other	17 (19.5)
**Marital status**	
Widowed	47 (54.0)
Never married	16 (18.4)
Married	14 (16.1)
Divorced or separated	10 (11.5)
**Primary life-limiting diagnosis**	
Dementia	44 (50.6)
Frailty	15 (17.2)
Other	28 (32.2)
**Number of comorbidities**	
Mean (SD)	4 (1.5)
1 to 2	18 (20.7)
3	22 (25.3)
4	20 (23.0)
5	17 (19.5)
$\:\ge\:$6	10 (11.5)
**Comorbidities**	
Hypertension	52 (59.8)
Dementia	51 (58.6)
Depression	35 (40.2)
Stroke or transient ischemic attack	23 (26.4)
Diabetes	21 (24.1)
Non-metastatic cancer	19 (21.8)
Hypothyroidism	15 (17.2)
Atrial fibrillation	14 (16.1)
Chronic pulmonary disease	14 (16.1)
Chronic heart failure	13 (14.9)
Chronic kidney disease	9 (10.3)
Chronic pain	8 (9.2)
Myocardial infarction	8 (9.2)
Parkinson’s disease	8 (9.2)
**Palliative Performance Scale**	
40 to 50%	35 (40.2)
30%	42 (48.3)
10 to 20%	10 (11.5)
**CHESS score**	
**Time (days) CHESS score prior to consultation**,** mean (SD)**	37 (25.7)
**CHESS score prior to initial consultation**	
0	25 (28.7)
1	24 (27.6)
2	20 (23.0)
3	9 (10.3)
4 to 5	9 (10.3)
**Substitute decision maker**	
Children	54 (62.1)
Spouse or partner	11 (12.6)
Siblings	10 (11.5)
Any other relative, friend, or Public Guardian and Trustee	12 (13.8)

N (%), unless otherwise specified. CHESS: Changes in Health, End-Stage Disease and Signs and Symptoms scale, LTC: Long-term care

All LTC residents referred to the palliative care consultation team were seen. Most residents were seen once (55.2%), while 25.3% were seen for one follow-up visit, and 19.5% were seen for two or more in follow-up; median one visit (IQR 1–2). Almost all the initial consultations were seen in-person. Some of the follow-up visits were seen virtually either by telephone or video (21.8%). See Table 2 for details.

**Table 2:** Access to the palliative care consultation team in long-term care

Access to palliative care consultation team	LTC residents (*n* = 87)
**Number of times seen**,** median (IQR)**	1 (1–2)
**One consultation only**	48 (55.2)
**Number of follow-up visits (if required)**	
1	22 (25.3)
$\:\ge\:$2	17 (19.5)
**Modality of palliative care visit**	
**Number of in-person visits per resident**	
1	51 (58.6)
2	19 (21.8)
$\:\ge\:$3	14 (16.1)
**Number of virtual visits per resident**	
$\:\ge\:$1 telephone visit	9 (10.3)
$\:\ge\:$1 video visit	11 (12.6)
$\:\ge\:$1 telephone or video visit	19 (21.8)
**Survival time (days) from initial consultation**, ***n*** **= 53**	
Median survival time (IQR)	76 (21–263)
0 to < 30 days	15 (28.3)
30 to < 90 days	16 (30.2)
$\:\ge\:$90 days	22 (41.5)

N (%), unless otherwise specified. IQR: Interquartile range, LTC: Long-term care

Among residents that died during the study period (*n* = 53), the median survival time was 76 (IQR 21–263) days. 25% of the residents were referred to palliative care more than 90 days prior to death. On the other hand, most of the residents (58.5%) were referred with less than 90 days of survival time. See Table 3 for details.

**Table 3:** Outcomes on advance care planning, hospital transfers, and disposition among long-term care residents referred to palliative care

Outcomes	LTC residents (*n* = 87)
**Advance care plan with “Transfer to Hospital”,*n*** **= 41**	
Transitioned to “Do Not Transfer” after consultation	22 (53.7)
**“Full Code” status**, ***n*** **= 17**	
Transitioned to “Do Not Resuscitate” after consultation	13 (76.5)
**Number of hospital transfers per resident**	
None	68 (78.2)
1 to 2	19 (21.8)
**Median (IQR) of hospital transfers per year**, ***n*** **= 19**	1 (1–4)
**Disposition**	
Alive	34 (39.1)
Died	53 (60.9)

N (%), unless otherwise specified. LTC: Long-term care

Among residents with an advance care plan with documentation of “Transfer to Hospital” (*n* = 41), 53.7% were agreeable to transition their advance care plan to “Do Not Transfer” and receive supportive care in LTC. For residents with “Full Code” status prior to palliative care involvement (*n* = 17), 76.5% of them transitioned to a “Do Not Resuscitate” status afterwards. 22% of residents had one or two hospital transfers after the initial palliative care consultation, a median of one hospital transfer per year among residents with at least one hospital transfer (IQR 1–4). The hospitalisation rate was one per 1000 resident-year. Among the 60.9% of residents referred to palliative care that died during the study period, almost all residents died at the home. Due to the small number of in-hospital deaths ($\:\le\:$6), they are not reported here.

## Discussion

### Key findings

The findings of this study provide information about a palliative care consultation team that serves a medically complex and frail older adult population residing in a single LTC home. Many residents who were referred to palliative care at this LTC home had multimorbidity, along with a primary life-limiting diagnosis of dementia and/or frailty. Most of these residents had a PPS of 30% or greater with low CHESS scores, indicating low levels of health instability. Approximately 40% of the residents who died during the study period accessed palliative care earlier than the last three months of life. These findings underscore the importance of supporting LTC residents with serious, life-limiting, and typically non-malignant diseases by integrating palliative care consultation teams. These teams can provide direct patient care in LTC settings when needed. This approach has the potential to facilitate symptom management and advance care planning for residents with complex palliative care needs, reduce hospitalisations, and support residents to die in place, if that is their preference.

### What this study adds

Previous research has identified four main types of palliative care programs in LTC homes: (1) referrals to palliative care consultation teams for end-of-life care only, (2) end-of-life care in hospice-like units, (3) capacity-building activities to enhance primary palliative care, and (4) capacity building facilitated by palliative care teams without their direct patient care [16]. The findings of this study highlight the utility of referring to palliative care consultation teams to address palliative needs before end of life, particularly for non-malignant diseases such as dementia and frailty, which have similar pain and symptom burdens as cancer [17–19]. The integration of palliative care teams in LTC also has the potential to decrease unnecessary and avoidable hospital transfers. We report a relatively lower rate of hospital transfers among LTC residents receiving palliative care, one hospitalisation per 1000 resident-year in our study compared to 358 per 1000 resident-year among residents with similar demographics in not-for-profit LTC facilities across Ontario [20]. Our findings demonstrate the potential for palliative care teams to deliver resident-centred care tailored to their last months to years of life. This approach may also make acute care beds available for those in need, thereby optimizing the use of system resources. However, future comparative studies between LTC homes are warranted to further examine these findings.

For LTC residents with complex palliative care needs, it is not ideal to make late referrals to palliative care consultation teams. Crisis management plans and end-of-life care plan decisions should be made well in advance to ensure the resident’s wishes, values, and beliefs are respected [21]. Having conversations about what is important to the resident before they reach the end of life ensures that they are prepared for decision-making when the time comes [22, 23]. Palliative care consultation teams can also play a critical role in training, driving cultural change, and enhancing meaningful conversations about advance care planning that are led by the primary care team. This ensures that residents and their care partners are ready to engage in discussions about what matters most to them [16]. 

### Limitations

The results of this study should be considered in context of its limitations. Firstly, we present an observational single-centre cohort study that is limited in its generalizability. Nevertheless, this study establishes a foundation for future multicentre comparative research on palliative consultation teams in LTC. Secondly, the prevalence of comorbidities may be underreported in resident medical records. For example, chronic kidney disease is likely to be underreported when compared to national Canadian data on the prevalence of chronic kidney disease among older adults [24]. However, major comorbidities that impact resident quality of life since admission into LTC are likely to be captured. Thirdly, the CHESS scores are likely to be underestimated as they were collected from available records prior to initial palliative care consultation and not determined on the day of consultation. However, the mean days between the CHESS score and initial consultation was only about a month apart, which still informs the relative instability of those referred to palliative care. Finally, we did not examine pain, symptom, psychosocial, and caregiver needs as part of this study. We also did not examine the knowledge, skills, or perspectives of LTC staff. In this study, we focused on describing LTC residents and their outcomes after referral to palliative care. Further research is required to understand the effect of integrated palliative care consultations teams in LTC homes on symptom management, psychosocial supports, and internal capacity of LTC staff to deliver a primary palliative approach to care.

## Conclusions

We report on an original retrospective cohort study of a palliative care consultation team for LTC residents in Toronto, Ontario. The findings of this study highlight the potential benefits of integrating palliative care consultation teams within a LTC home, particularly for residents with non-malignant life-limiting diagnoses. The findings of this study also suggest that such models of care can improve the delivery of palliative care, facilitate advance care planning discussions, and reduce hospital transfers. The insights gained from this study provide a foundation for future implementation research aimed at enhancing palliative care delivery in LTC homes.

## Data Availability

The dataset generated and analysed during the current study is not publicly available to safeguard individual privacy.

## Abbreviations

LTC: Long-term care
CHESS: Changes in health, end-stage disease and signs and symptoms

## References

[1] Statistics Canada. Population Projections for Canada, Provinces and Territories, 2009 to 2036. Ottawa: Statistics Canada; 2010 Jun [cited 2025 Jan 29]. Report No.: Catalogue no. 91-520-X. Available from: https://publications.gc.ca/collections/collection_2010/statcan/91-520-X/91-520-x2010001-eng.pdf

[2] Sheets DJ, Gallagher EM. Aging in Canada: state of the Art and science. Gerontologist. 2013;53(1):1–8. 23197394 10.1093/geront/gns150

[3] Canadian Institute for Health Information. Access to palliative care in Canada, 2023. Ottawa, ON: CIHI. 2023 [cited 2024 Apr 25]. Available from: https://www.cihi.ca/sites/default/files/document/access-to-palliative-care-in-canada-2023-report-en.pdf

[4] Kruizinga J, Lucchese S, Vellani S, Rivas VM, Shamon S, Diedrich K, et al. Perspectives across Canada about implementing a palliative approach in long-term care during COVID-19. BMC Palliat Care. 2023;22(1):32. 36991407 10.1186/s12904-023-01142-3 PMC10060130

[5] Brazil K, Bédard M, Krueger P, Taniguchi A, Kelley ML, McAiney C, et al. Barriers to providing palliative care in long-term care facilities. Can Fam Physician. 2006;52(4):472–3. 17327890 PMC1481676

[6] Liu X, Chang YC, Hu WY. The effectiveness of palliative care interventions in Long-Term care facilities: A systematic review. J Pers Med. 2024;14(7):700. 39063954 10.3390/jpm14070700 PMC11277722

[7] Sussman T, Kaasalainen S, Mintzberg S, Sinclair S, Young L, Ploeg J, et al. Broadening End-of-Life comfort to improve palliative care practices in long term care. Can J Aging. 2017;36(3):306–17. 28747236 10.1017/S0714980817000253

[8] Froggatt KA, Moore DC, Van den Block L, Ling J, Payne SA, PACE consortium collaborative authors on behalf of the European Association for Palliative Care. Palliative care implementation in Long-Term care facilities: European association for palliative care white paper. J Am Med Dir Assoc. 2020;21(8):1051–7. 32115370 10.1016/j.jamda.2020.01.009

[9] Comart J, Mahler A, Schreiber R, Rockett C, Jones RN, Morris JN. Palliative care for long-term care residents: effect on clinical outcomes. Gerontologist. 2013;53(5):874–80. 23220397 10.1093/geront/gns154

[10] Miller SC, Dahal R, Lima JC, Intrator O, Martin E, Bull J, et al. Palliative care consultations in nursing homes and End-of-Life hospitalizations. J Pain Symptom Manage. 2016;52(6):878–83. 27650008 10.1016/j.jpainsymman.2016.05.017 PMC5154868

[11] Tonelli M, Wiebe N, Fortin M, Guthrie B, Hemmelgarn BR, James MT, et al. Methods for identifying 30 chronic conditions: application to administrative data. BMC Med Inf Decis Mak. 2015;15:31. 10.1186/s12911-015-0155-5 PMC4415341 25886580

[12] Baik D, Russell D, Jordan L, Dooley F, Bowles KH, Masterson Creber RM. Using the palliative performance scale to estimate survival for patients at the end of life: A systematic review of the literature. J Palliat Med. 2018;21(11):1651–61. 30129809 10.1089/jpm.2018.0141 PMC6211821

[13] Canadian Institute for Health Information. interRAI CA (IRRS). Calculating the CHESS Scale Score. Ottawa, ON: CIHI; 2022 Jul [cited 2025 Jan 29]. Available from: https://www.cihi.ca/sites/default/files/document/interrai-ca-irrs-calculating-chess-scale-score-jobaid-en.pdf

[14] Hospice Palliative Care Ontario. Advanced Care Planning Ontario: Who is My Substitute Decision Maker (SDM)?. 2024 [cited 2024 Mar 2]. Available from: https://advancecareplanningontario.ca/substitue-decision-makers/who-is-my-sdm

[15] Murmann M, Manuel DG, Tanuseputro P, Bennett C, Pugliese M, Li W, et al. Estimated mortality risk and use of palliative care services among home care clients during the last 6 months of life: a retrospective cohort study. CMAJ. 2024;196(7):E209–21. 38408785 10.1503/cmaj.221513 PMC10896599

[16] Kaasalainen S, Sussman T, McCleary L, Thompson G, Hunter PV, Wickson-Griffiths A, et al. Palliative care models in Long-Term care: A scoping review. Nurs Leadersh (Tor Ont). 2019;32(3):8–26. 31714204 10.12927/cjnl.2019.25975

[17] Fox S, FitzGerald C, Harrison Dening K, Irving K, Kernohan WG, Treloar A, et al. Better palliative care for people with a dementia: summary of interdisciplinary workshop highlighting current gaps and recommendations for future research. BMC Palliat Care. 2017;17(1):9. 28705196 10.1186/s12904-017-0221-0 PMC5512895

[18] Sachs GA, Shega JW, Cox-Hayley D. Barriers to excellent End-of-life care for patients with dementia. J Gen Intern Med. 2004;19(10):1057–63. 15482560 10.1111/j.1525-1497.2004.30329.x PMC1492583

[19] Stow D, Spiers G, Matthews FE, Hanratty B. What is the evidence that people with frailty have needs for palliative care at the end of life? A systematic review and narrative synthesis. Palliat Med. 2019;33(4):399–414. 30775957 10.1177/0269216319828650 PMC6439946

[20] Tanuseputro P, Chalifoux M, Bennett C, Gruneir A, Bronskill SE, Walker P, et al. Hospitalization and mortality rates in Long-Term care facilities: does For-Profit status matter?? J Am Med Dir Assoc. 2015;16(10):874–83. 26433862 10.1016/j.jamda.2015.06.004

[21] Zimmermann C, Swami N, Krzyzanowska M, Hannon B, Leighl N, Oza A, et al. Early palliative care for patients with advanced cancer: a cluster-randomised controlled trial. Lancet. 2014;383(9930):1721–30. 24559581 10.1016/S0140-6736(13)62416-2

[22] The Canadian Nurses Association, the Canadian Hospice Palliative Care Association, and the Canadian Hospice Palliative Care Nurses Group. Joint Position Statement - The palliative approach to care and the role of the nurse. 2015 [cited 2024 Apr 25]. Available from: https://www.virtualhospice.ca/Assets/A Palliative Approach to Care and the Role of the Nurse_20151001151135.pdf

[23] Heckman GA, Boscart V, Quail P, Keller H, Ramsey C, Vucea V, et al. Applying the Knowledge-to-Action framework to engage stakeholders and solve shared challenges with Person-Centered advance care planning in Long-Term care homes. Can J Aging / La Revue Canadienne Du Vieillissement. 2022;41(1):110–20. 10.1017/S0714980820000410 33583447

[24] Bello AK, Ronksley PE, Tangri N, Kurzawa J, Osman MA, Singer A, et al. Prevalence and demographics of CKD in Canadian primary care practices: A Cross-sectional study. Kidney Int Rep. 2019;4(4):561–70. 30993231 10.1016/j.ekir.2019.01.005 PMC6451150

